# Sorafenib in patients with advanced biliary tract carcinoma: a phase II trial

**DOI:** 10.1038/sj.bjc.6605458

**Published:** 2009-11-24

**Authors:** C Bengala, F Bertolini, N Malavasi, C Boni, E Aitini, C Dealis, S Zironi, R Depenni, A Fontana, C Del Giovane, G Luppi, P Conte

**Affiliations:** 1 Division of Medical Oncology, University Hospital, University of Modena and Reggio Emilia, Modena, Italy; 2 Division of Medical Oncology, S. Maria Nuova Hospital, Reggio Emilia, Italy; 3 Division of Medical Oncology, Carlo Poma Hospital, Mantova, Italy

**Keywords:** biliary tract cancer, phase II trial, sorafenib

## Abstract

**Background:**

Advanced biliary tract carcinoma has a very poor prognosis, with chemotherapy being the mainstay of treatment. Sorafenib, a multikinase inhibitor of VEGFR-2/-3, PDGFR-*β*, B-Raf, and C-Raf, has shown to be active in preclinical models of cholangiocarcinoma.

**Methods:**

We conducted a phase II trial of single-agent sorafenib in patients with advanced biliary tract carcinoma. Sorafenib was administered at a dose of 400 mg twice a day. The primary end point was the disease control rate at 12 weeks.

**Results:**

A total of 46 patients were treated. In all, 26 (56%) had received chemotherapy earlier, and 36 patients completed at least 45 days of treatment. In intention-to-treat analysis, the objective response was 2% and the disease control rate at 12 weeks was 32.6%. Progression-free survival (PFS) was 2.3 months (range: 0–12 months), and the median overall survival was 4.4 months (range: 0–22 months). Performance status was significantly related to PFS: median PFS values for ECOG 0 and 1 were 5.7 and 2.1 months, respectively (*P*=0.0002). The most common toxicities were skin rash (35%) and fatigue (33%), requiring a dose reduction in 22% of patients.

**Conclusions:**

Sorafenib as a single agent has a low activity in cholangiocarcinoma. Patients having a good performance status have a better PFS. The toxicity profile is manageable.

Adenocarcinoma of the gallbladder and cholangiocarcinoma account for 4 and 3% of all gastrointestinal cancers, respectively (Fong *et al*, 2001, p 1178). The incidence is 1–2 per 100 000 in the United States and Europe, and increases to 87 per 100 000 in Southeast Asia (De Groen *et al*, 1999). These are highly fatal malignancies, with 1- and 2-year survival rates of 25 and 13%, respectively (Fong *et al*, 1996; Lazcano-Ponce *et al*, 2001).

Surgery represents the only curative option; however, only ∼25% of patients with gallbladder carcinoma are radically resectable, with a 5-year survival rate of ∼40% patients with cholangiocarcinoma have an even worse prognosis, with a 5-year survival rate of 5–10%. Patients with unresectable disease receive palliative chemotherapy. Many agents including fluoropyrimidines, gemcitabine, cisplatin/oxaliplatin, mitomycin C, doxorubicin, docetaxel, and irinotecan have been tested with response rates ranging from 10 to 40% (Berardi *et al*, 2006; Hezel and Zhu, 2008). However, despite its limited activity and morbidity, chemotherapy remains the mainstay of treatment for most patients and the combination of gemcitabine plus cisplatin is recommended as the standard of care. (Eckel and Schmid, 2007; Valle *et al*, 2009).

Molecular alterations such as disruption of the MAPK pathway and activating RAS and B-Raf mutations have been described in these tumours, and these molecular abnormalities may constitute a target for new biological agents (Tannapfel *et al*, 2003). However, biliary tract carcinoma usually shows hypovascularity, suggesting that angiogenesis pathways may have a minor role in the pathogenesis and progression of disease (Kawahara *et al*, 1998).

Sorafenib, an orally available multikinase inhibitor of VEGFR-2/-3, PDGFR-*β*, B-Raf, C-Raf, FLT-3, and RET, has shown an anti-tumour activity in preclinical models of breast, colon, and pancreatic cancer, as well as in a phase I clinical trial in solid tumours (Wilhelm *et al*, 2004; Beeram *et al*, 2005; Strumberg *et al*, 2005) and in a phase II trial in metastatic thyroid cancer (Kloos *et al*, 2009). On the basis of randomised clinical trials, sorafenib has been approved for the treatment of renal cell carcinoma and hepatocellular carcinoma (Escudier *et al*, 2007; Llovet *et al*, 2008a). More recently, Huether *et al* (2007) have demonstrated that sorafenib can potently suppress the growth of human cholangiocarcinoma cells in a preclinical model.

On these premises, we have designed a phase II clinical trial of a single-agent sorafenib in patients with advanced biliary tract carcinoma. Moreover, we planned to perform biomarker analysis including the BRAF mutation and VEGFR-2 expression. These analyses are still ongoing and will be presented in future research.

## Patients and methods

### Patient selection

Patients were eligible if they had a pathologically proven diagnosis of advanced and unresectable cholangiocarcinoma, extrahepatic biliary duct carcinoma, or gallbladder carcinoma, an ECOG performance status 0–1, measurable disease, as well as adequate liver and renal function tests. A total serum bilirubin level up to 3.0 mg per 100 ml was allowed. Advanced disease was defined as primary tumours or relapsed disease not amenable for radical surgery or metastatic disease. Earlier treatments including surgery, locoregional treatment, and systemic therapy were allowed as well. Patients were required to sign an informed consent, and the Local Ethics Committee of the Province of Modena approved the study.

### Study design

This was a phase II single-arm open-label non-randomised multicentre trial. The primary objective was to evaluate the activity, defined as the disease control rate at 12 weeks, of sorafenib as a single agent in patients with advanced cholangiocarcinoma. Disease control rate was defined as the percentage of patients without disease progression (complete response, partial response, stable disease) and still on treatment at 12 weeks. Secondary objectives included progression-free survival (PFS), overall survival (OS), and tolerability.

Progression-free survival was defined as the time from the first day of administration of the study drug to disease progression or death for toxicity or disease progression.

Overall survival was defined as the time from the first day of study medication administration to death or last contact.

### Treatment plan

Sorafenib was administered at a fixed dose of 400 mg twice a day continuously in a 4-week cycle until disease progression, unacceptable toxicity, physician's decision to remove the patient, or withdrawal of patient consent.

Doses were delayed or reduced in case of haematological or non-haematological toxicity graded according to the NCI Common Toxicity Criteria Version 2.0. The sorafenib predefined dose level reductions were level −1: 200 mg po q 12 h; and level −2: 200 mg po per day. In case of G1 toxicity or first occurrence of G2 toxicity lasting <7 days, no dose modification or delay was planned. For G2 toxicity lasting >7 days, for the second and third occurrence of G2 or G3 toxicity, a treatment delay until resolution to G0–1 and a dose reduction to level −1 were planned. After the fourth occurrence, treatment was discontinued. In case of G3 non-haematological or G4 haematological toxicity at first and second occurrence, treatment was delayed until toxicity resolution to G0–1, and 1 level dose reduction was planned. After the third occurrence, treatment was discontinued. In case of G4 toxicity, treatment was delayed until toxicity resolution, and a dose reduction of 2 dose levels was planned. If, after a 3-week delay, the patient did not recover to G0–1, treatment was discontinued.

### Assessment of disease

Baseline evaluation included medical history, physical examination, and tumour assessment with computed tomography (CT) or magnetic resonance (MRI) within 28 days of study entry; CT/MRI scans of the brain and bone scans were performed if clinically indicated. Tumour response was evaluated every 12 weeks (3 cycles) with the same imaging techniques (CT or MRI) used at baseline. In case of complete, partial response, or stable disease, a reassessment of disease was performed using the same technique (CT or MRI) after 4–6 weeks to confirm the response.

### Statistical analysis

The primary end point of the trial was the disease control rate (CR+PR+SD according to the RECIST criteria) at 12 weeks. Sample size was calculated according to the Simon double-stage design test. We assumed as a non-interest hypothesis, a disease control rate ⩽0.15 and, as result of interest, a disease control rate ⩾0.35. According to our hypothesis, the study required 32 subjects. If the number of patients with disease control was ⩾8 or more, the hypothesis that *P*⩽0.150 would have been rejected with a target error rate of 0.100 and an actual error rate of 0.096. If the number of events was ⩽7 or less, the hypothesis that *P*⩾0.350 would have been rejected with a target error rate of 0.100 and an actual error rate of 0.082.

## Results

### Patient characteristics

From August 2006 to December 2007, 46 patients with recurrent or metastatic biliary tract adenocarcinoma were enrolled into this phase II trial; enrolment was expanded to 46 patients because 14 patients discontinued the treatment within 45 days from study entry. The main patient characteristics were as follows: median age 66 years (37–80); 32 cholangiocarcinomas (69.6%), 14 gallbladder carcinomas (30.4%), and all patients were not amenable to surgery. A total of 26 patients had received chemotherapy earlier, including gemcitabine, platinum compound, and fluoropyrimidines (Table 1).

### Toxicity

Toxicity was evaluated on the entire group of 46 patients. There were no treatment-related deaths. In all, 10 patients (21.7%) had a dose reduction: 1 to 75% 9 to 50%. These dose reductions were due to G3 toxicity: skin rash in six patients; HFS in two patients; and fatigue and mucositis in two patients. Eight patients (17.4%) discontinued the treatment because of toxicity: three patients for prolonged (>3 weeks) G3 hand–foot syndrome, one patient for prolonged G3 skin rash, one for G3 diarrhoea, one for G3 cardiac ischaemia, one for prolonged G3 fatigue, and one for prolonged G2 thrombocytopaenia.

The most common event was skin rash that occurred in 16 patients (35%), which was classified as G1–2 in 9 patients (20%) and as G3 in 7 patients (15%). Eight patients (17%) presented hand–foot syndrome: G1–2 in three patients (37.5%) and G3 in five patients (11%). Liver dysfunction was observed in 28% of patients, mostly due to cancer progression. However, G1–2 and G3 drug-related liver toxicity was observed in two (4.3%) and one patient (2.2%), respectively. Other G3 toxicities included fatigue and diarrhoea in five patients (11%) and in one patient (2%), respectively. Three patients experienced deep venous thrombosis (DVT) and two had pulmonary embolism. One patient taking corticosteroids for concomitant chronic obstructive pulmonary disease and paraneoplastic fever experienced a G4 gastrointestinal bleeding. Haematological toxicity was mild: G1–2 thrombocytopaenia was observed in 13 patients (28%) and G3 anaemia in 1 patient (2.2%) Table 2 shows the adverse events that are considered to be possibly drug related.

### Outcome

According to the intention-to-treat analysis, an analysis for the primary end point (DCR) was performed on the entire group of patients (46 patients): 1 patient achieved a partial response (2.2%) and 14 patients (30.4%) achieved a stable disease for a disease control rate of 32.6% at 12 weeks. The disease control rates in the chemonaive and pretreated patients were 30 and 35%, respectively. In all, 14 patients received <45 days of treatment (median: 22 days; range: 5–37): 1 patient for low compliance, 5 for early toxicity, and 8 for very early progression. However, for the intent-to-treat analysis, the 14 patients were considered as having disease progression. The median duration of disease control in the group of patients receiving at least 12 weeks of treatment was 6 months (3.5–11.9).

**Figure 1:**
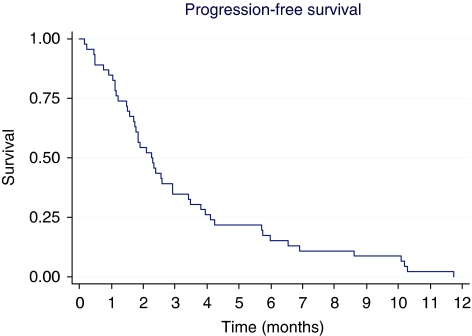
Progression-free survival (*N*=46).

**Figure 2:**
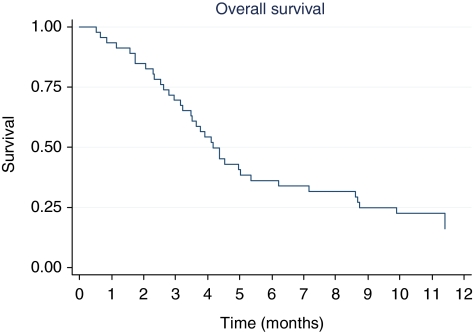
Overall survival (*N*=46).

**Figure 3:**
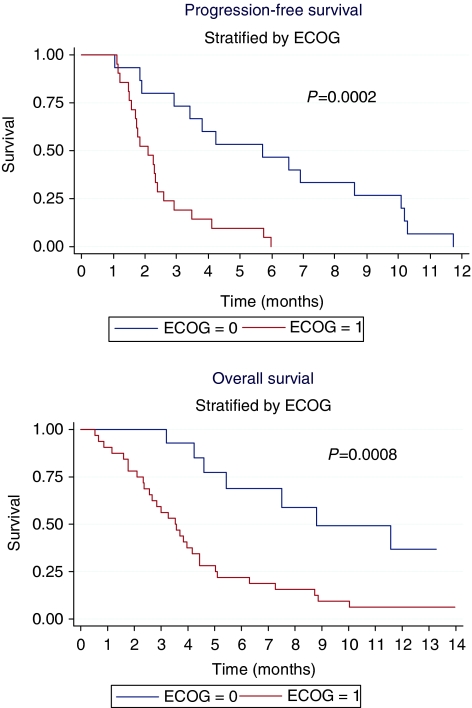
Progression-free survival and overall survival stratified by ECOG performance status.

According to the intention-to-treat analysis, the median PFS was 2.3 months (range: 0–12 months) (Figure 1) and median OS was 4.4 months (range: 0–22 months) (Figure 2). Neither disease site (intra *vs* extrahepatic) nor previous treatments were significantly related to PFS. On the contrary, PFS was significantly related to performance status: median PFS was 5.7 for ECOG 0 and 2.1 months for ECOG 1 (*P*=0.0002). This advantage was also observed in OS: median OS was 8.8 months (range: 3.2–13.3) for ECOG 0 and 3.5 months (0.53–14.0) for ECOG 1 (*P*=0.0008) (Figure 3).

## Discussion

Very few treatment options are available for advanced biliary tract carcinoma, and the prognosis of these patients is very poor. Recently, a pooled analysis of 112 clinical trials of chemotherapy in advanced biliary tract carcinoma showed a tumour response rate and disease control rate of 22.6 and 57.3%, respectively, whereas the median time to progression and OS were 4.1 and 8.1 months, respectively (Eckel and Schmid, 2007). Recently, the combination of gemcitabine and cisplatin has been reported to be significantly superior than gemcitabine alone in patients with advanced or metastatic advanced biliary tract cancer (Furuse *et al*, 2009; Valle *et al*, 2009).

Owing to the molecular alterations described in cholangiocarcinomas, we designed a phase II trial to test the activity of sorafenib as a single agent in advanced and metastatic biliary tract carcinoma.

Single-agent sorafenib has very low activity (measured as an objective response) in renal cell and hepatocellular carcinoma; however, it can significantly prolong PFS and OS in both tumour types. Therefore, we have identified the disease control rate at 12 weeks as the primary end point of our trial. This is a suitable end point in oncology when a non-cytotoxic agent is used and a low objective response rate, along with a high stable disease rate, is expected (Llovet *et al*, 2008b; Adjei *et al*, 2009).

At the intention-to-treat analysis, the response rate and the stable disease rate were 2.2 and 30.4%, respectively, with a disease control rate of 32.6%. Unfortunately, the median time to disease progression and OS were disappointing: 2.3 and 4.4 months, respectively. However, patients with an ECOG 0 performance status had a median PFS and a median OS that were significantly longer than did patients with an ECOG 1 performance status: 5.7 *vs* 2.1 months and 8.8 *vs* 3.5 months, respectively. To our knowledge, very few data on sorafenib in cholangicarcinoma have been reported so far. LaRocca *et al* (2007) have reported a long-lasting stable disease in two patients with advanced cholangiocarcinoma. El-Khoureiry *et al* have studied sorafenib as first-line treatment in advanced and metastatic gallbladder and cholangiocarcinoma in a phase II trial. They reported a disease control rate of 35% with a median time to progression and a median OS of 2 and 6 months, respectively; grade 3–4 toxicities were observed in 20 patients (66.7%), and 1 patient died because of cardiovascular toxicity (El-Khoureiry *et al*, 2007). These data, as well as our data, show that the activity of sorafenib as a single agent is marginal compared with systemic chemotherapies using gemcitabine and cisplatin; moreover, patients with very good performance status may benefit from this treatment.

The toxicity profile of sorafenib in our trial was slightly different from that reported by Llovet *et al* (2008a)in patients with hepatocellular carcinoma treated with sorafenib. In particular, we observed more frequent skin rash (35 *vs* 16%), liver toxicity (6.5 *vs* <1%), and fatigue (33 *vs* 22%). Moreover, five patients experienced vascular toxicity: DVT in three patients and pulmonary embolism in two patients. These toxicities were not observed in the SHARP study. A possible explanation for the higher occurrence of toxicity in our trial can be the inclusion of patients who had received previous treatment for advanced disease (54% of the patients were pretreated with chemotherapy). However, the safety profile of sorafenib in our trial is acceptable in comparison with that observed in patients treated with chemotherapy. Several phase II clinical trials of gemcitabine plus cisplatin in advanced biliary tract carcinoma reported a grade 3–4 toxicity in the range of 33–75% (Thongprasert *et al*, 2005; Iyer *et al*, 2007; Meyerhardt *et al*, 2008).

Very few data are available on the combination of sorafenib with chemotherapeutic agents. Promising results have been reported with the combination of sorafenib with carboplatin and paclitaxel in melanoma and ovarian cancer, with oxaliplatin in gastric and colorectal cancer, and with capecitabine in different solid tumours (Takimoto and Awada, 2008). Although a direct comparison between the combination of sorafenib plus chemotherapy *vs* single agent is limited, a recent comprehensive review showed that a combination of sorafenib with cytotoxic agents is generally well tolerated (Takimoto and Awada, 2008). Moreover, the toxicity profiles observed in combination trials indicate that toxicities associated with sorafenib rarely overlap with those induced by chemotherapy (Dal Lago *et al*, 2008; Takimoto and Awada, 2008). Finally, bevacizumab, a humanised anti-VEGF-A monoclonal antibody, has been tested in patients with biliary tract cancer, in combination with gemcitabine and oxaiplatin, in a phase II trial. A total of 19 patients have been treated with a partial response rate of 15.8% and with a stable disease rate of 26.3% (Clark *et al*, 2007). Other anti-angiogenic agents including vandetanib and cediranib are currently under evaluation in ongoing clinical trials.

In conclusion, our study shows that sorafenib has a low activity in cholangiocarcinoma. However, patients having a very good performance status may experience some benefit. In future studies, a combination of sorafenib with cytotoxic drugs could be tested. Correlative studies to define predictive molecular markers for sorafenib should be undertaken.

## Figures and Tables

**Table 1:** Patient characteristics

Number of patients	(*n*=46)
Median age, years	66 (range: 37–80)
	
*Gender*	*n* (%)
Male	20 (44)
Female	26 (56)
	
*ECOG performance status*	
ECOG 0	15 (33)
ECOG 1	31 (67)
	
*Site*	
Gallbladder	14 (30)
Extrahepatic	5 (10)
Intrahepatic	27 (60)
	
*TNM stage*	
II	2 (4)
III	7 (15)
IV	37 (81)
	
*Previous treatments*	
Surgery	28 (61)
Radical	19 (41)
Palliative/biopsy	9 (20)
Chemotherapy lines	26 (56)
1	6 (13)
2	11 (24)
3	7 (15)
>3	2 (4)
Radiotherapy	5 (10)
Stereotactic	1 (2)
Palliative	4 (8)
Local treatments	4 (8)
Hyperthermy	2 (4)
RF	1 (2)
TACE/RF	1 (2)

Abbreviations: ECOG=Eastern Cooperative Oncology Group; TNM=tumour–node–metastasis; TACE=transcatheter arterial chemoembolisation; RF=radio frequency ablation.

**Table 2:** Toxicity (*n*=46)

	**G1–G2 (%)**	**G3–G4 (%)**	**Total (%)**
Skin rash	9 (20)	7 (15)	16 (35)
Hand–foot syndrome	3 (6)	5 (11)	8 (17)
			
*Gastrointestinal*	18 (39)	7 (15)	25 (54)
Diarrhoea	3 (6)	1 (2)	4 (8)
Stomatitis	4 (8)	0 (0)	4 (8)
Nausea/vomiting	4 (8)	0 (0)	0 (8)
Liver enzyme	2 (4)	1 (2)	3 (6)
			
*Haematological*	13 (28)	1 (2)	14 (30)
Anaemia	2 (4)	1 (2)	3 (6)
Trombocytopaenia	11 (24)	0 (0)	11 (24)
Neutropaenia	0 (0)	0 (0)	0 (0)
Febrile neutropaenia	0 (0)	0 (0)	0 (0)
			
*Systemic symptoms*	10 (22)	5 (11)	2 (33)
Fatigue	10 (22)	5 (11)	15 (33)
Infection	0 (0)	1 (2)	1(2)
			
*Cardiovascular*	1 (2)	3 (6)	4 (8)
Deep vein thrombosis	1 (2)	0 (0)	1 (2)
Pulmonary embolism	0 (0)	2 (4)	2 (4)
Cardiac ischaemia	0 (0)	1 (2)	1 (2)
Coagulation (DIC)	1 (2)	0 (0)	1 (2)
Haemorrhage/bleeding	0 (0)	1 (2)	1 (2)
Metabolic/laboratory	5 (11)	1 (2)	6 (13)

Abbreviation: DIC=disseminated intravascular coagulation.
